# Perspective of Monitoring Heavy Metals by Moss Visible Chlorophyll Fluorescence Parameters

**DOI:** 10.3389/fpls.2019.00035

**Published:** 2019-01-25

**Authors:** Yang-Er Chen, Nan Wu, Zhong-Wei Zhang, Ming Yuan, Shu Yuan

**Affiliations:** ^1^College of Life Science, Sichuan Agricultural University, Ya’an, China; ^2^College of Resources, Sichuan Agricultural University, Chengdu, China

**Keywords:** chlorophyll fluorescence, moss, abiotic stress, heavy metal monitoring, non-photochemical quenching

## Abstract

Chlorophyll fluorescence measurements have been mainly applied to investigate the functioning of the photosynthetic apparatus in the diagnosis of environmental stress. Moss is sensitive to several abiotic stresses and is considered an environmental indicator. Therefore, moss chlorophyll fluorescence can be as a visual parameter applicable for monitoring heavy metal contaminants in water. Different from previous studies with value changes of chlorophyll fluorescence in mosses, we suggest that phenotypes with anthocyanin accumulation pattern and chlorosis pattern and colors of chlorophyll fluorescence images of the maximum efficiency of PSII photochemistry (Fv/Fm) and the quantum yield of PSII electron transport (Φ_PSII_) could reflect metal species groups and concentrations roughly. And we further indicated that Cr(III) and Cr(VI) could be monitored distinguishably according to the non-photochemical quenching (NPQ) fluorescence of sporadic purple and sporadic lavender images, respectively. It is interesting that the fluorescence color patterns were nearly the same for all treatment concentrations. This perspective provides additional data of chlorophyll fluorescence changes in moss under cold, heat, salinity, high light or osmotic stress. Only heat stress and high light have significant effects on the fluorescence parameters of Fv/Fm and Φ_PSII_. In contrast, mosses are less sensitive to short-term cold, salinity, and osmotic stress. While NPQ decreases rapidly under the osmotic stress. Nevertheless, heat stress, high light or osmotic stress does not usually co-occur in the place where the moss grows. Estimation through moss chlorophyll fluorescence color patterns is still a rapid and non-invasive method to monitor heavy metal pollutions in water.

## Introduction

As a result of global climate change, an increase in temperature, changes in precipitation pattern, stress by various environmental factors, alone or in combination, leads to a degradation of natural ecosystems (Hu et al., 2018). Evaluation of the physiological state of plants, their activity, monitoring the physical existence and intensity of environmental stress by operable methods can provide essential information for ecosystem management. Various experimental approaches and analytical methods have been developed for *in vivo* monitoring of plants’ physiological state, stress response, and tolerance, including RGB (red, green, and blue) imaging (Berger et al., 2010), thermal imaging (Jones et al., 2009), NIR (near-infrared) imaging (Seelig et al., 2009), and chlorophyll fluorescence (Kalaji et al., 2016). Among these methods, chlorophyll fluorescence, a powerful tool extensively used to analyze the status and function of photosystem II (PSII), is now being commonly employed in detecting the damage to photosynthetic apparatus caused by environmental stress (Stirbet et al., 2018). One of the main features of chlorophyll fluorescence measuring is non-invasive diagnostics, allowing researchers to get detailed information of plant performance without destructing the tested sample (Kalaji et al., 2014).

Chlorophyll fluorescence, which contains a large amount of information on PSII photochemistry reactions provides a measure of how well plants use the light energy absorbed at PSII for electron transport and is therefore a key measure of photosynthetic activity and performance (Baker, 2008). It is well known that many abiotic stresses cause changes in the photosynthetic apparatus at different metabolic levels (Pawłowicz et al., 2012) and environmental stress factors may produce directly or indirectly deleterious effects on PSII and many plant biochemical processes (Gururani et al., 2015). However, because photosynthesis is linked to many plant metabolic pathways, the alterations of photosynthesis can represent the physiological state of the plant and therefore chlorophyll fluorescence signal can reflect the influence of environmental factors on plants to some extent (Kalaji et al., 2016). Measuring chlorophyll fluorescence provides a rapid, non-destructive, and non-invasive screening tool for plant performance and the ability to cope with stress because perturbation of chlorophyll fluorescence in metabolism is sensitive (Barbagallo et al., 2003). Moreover, chlorophyll fluorescence along with other physiological parameters can bring more complex and useful information about the mechanisms contributing to stress responses (Chen et al., 2016, 2017, 2018c). We refer the reader to numerous other in-depth reviews on principles and method of chlorophyll fluorescence (Krause and Weis, 1991; Maxwell and Johnson, 2000; Baker, 2008; Murchie and Lawson, 2013).

At present, mosses and lichens have been used widely as bioindicators of atmospheric pollution (Pott and Turpin, 1996). Some mosses have also been used to evaluate water pollution (Bruns et al., 1997). In the late 1960s, Rühling and Tyler used mosses as sensitive bioindicators for surveying heavy metal contamination (Rühling and Tyler, 1968, 1970). Since then terrestrial mosses have been widely applied for pollution biomonitoring in different countries and regions (Pott and Turpin, 1996; Ermakova et al., 2004; Shotbolt et al., 2007; Chen et al., 2010). For example, using moss bags is a common strategy to measure heavy metals directly with terrestrial mosses. This technique employs placing suitable mosses, collected from clean areas, in a mesh net bag and measuring concentrations of contaminants in samples exposed to a polluted area (Ares et al., 2012). However, many methods using mosses for biomonitoring like moss bags are time-consuming, inconvenient and do not indicate the contaminants *in situ* immediately.

Moss rhizoid is too short to monitor deep soil pollution and therefore it is only used to monitor heavy metals in aquatic environment accurately. Based on the previous researches using chlorophyll fluorescence to detect abiotic stresses in the aquatic bioindicator mosses (Rau et al., 2007; Proctor and Smirnoff, 2011; Liepiņa and Ievinsh, 2013; Kangas et al., 2014; Jägerbrand and Kudo, 2016), we provided new ideas to monitor water heavy metals rapidly and non-invasively in a large-scale by moss visible parameters of Fv/Fm and Φ_PSII_ (Chen et al., 2015a). In other words, metal contaminations could be roughly estimated visually using the chlorophyll fluorescence images. This perspective provides further information about chlorophyll fluorescence changes in moss under cold, heat, salt stress, high light or osmotic stress. Only heat stress and high light had significant effects on Fv/Fm and Φ_PSII_. However, mosses are less sensitive to cold, salt stress or osmotic stress. While NPQ decreased rapidly under the osmotic stress. Fortunately, heat stress, high light, and osmotic stress do not usually co-occur in heavy-metal-polluted water. Moss fluorescence imaging is still a rapid and non-invasive method to monitor heavy metal contaminations in water. Moreover, insufficiency of this method and future research directions also are discussed.

## Moss as an Indicator Plant

Mosses are suitable objects for biomonitoring because of their extreme ability to accumulate pollutants such as heavy metals, radioisotopes and multifold chemical pollutants (Carballeira et al., 2006). The reasons for this over-accumulation capacity of mosses include the lack of the complex regulatory mechanisms of vascular plants due to the deficiency of a real root and vascular system (Tyler, 1990). In mosses, the leaves, which mostly lack a protective cuticle, normally have only a layer of cells and consequently offer a large surface for absorption (Ares et al., 2012). They readily absorb most of their nutrients and pollutants directly from atmosphere and precipitation through the entire surfaces (Tyler, 1990). As a result, Mosses can be applied to monitor heavy metals in aquatic environment, such as water, wetland, and moist soil surface. Mosses are more quickly and seriously poisoned by pollutants than vascular plants, and reflect with their specific victimization symptoms (Vanderpoorten and Goffinet, 2009).

## Biomonitoring Heavy Metal Contaminations by Chlorophyll Fluorescence Parameters in Mosses

**FIGURE 1:**
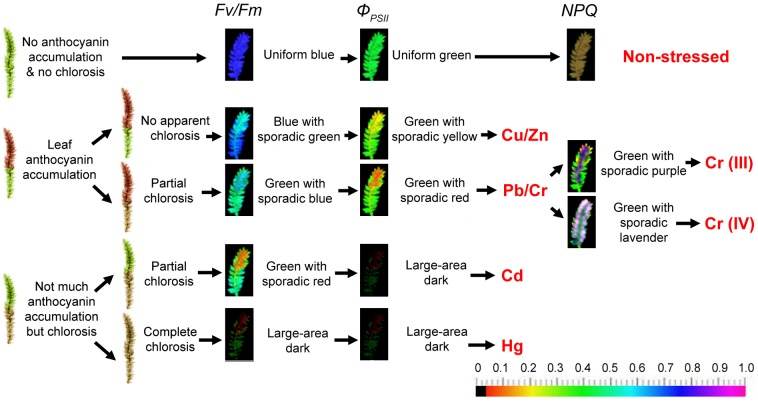
Putative heavy metal estimation criteria of moss phenotype and chlorophyll fluorescence imaging. The moss was cultured in distilled sterile modified Mohr medium (KNO_3_ 100 mg, CaCl_2_⋅4H_2_O 10 mg, MgSO_4_ 10 mg, KH_2_PO_4_ 136 mg, and FeSO_4_ 0.4 mg to 1000 mL distilled water, pH 7.5) for 3 days in lab before the metal stress to make them adjust to indoor environment (16/8 h photoperiod at 100 μmol of photons m^-2^ s^-1^, 25 ± 1°C). Metal stresses applied by adding 0 (control), 10, 25, or 50 μM CuSO_4_.5H_2_O, ZnSO_4_.7H_2_O, Pb(NO_3_)_2_, CrCl_3_, K_2_CrO_4_, CdCl_2_.2.5H_2_O, HgCl_2_ to the Mohr solution. Solutions were replaced every 2 days. The mosses were cultured or stressed for 30 days. Chlorophyll fluorescence images were obtained at room temperature using a modulated imaging fluorometer (the Imaging PAM *M*-Series Chlorophyll Fluorescence System, Heinz-Walz Instruments, Effeltrich, Germany) according to the instructions provided by the manufacturer. Images of Fv/Fm after dark adaptation and of Φ_PSII_ and NPQ at the steady-state of the induction curve with actinic illumination of 100 μmol photons m^-2^ s^-1^. The color scale shows at the bottom of the figure represents the range from 0 (black) to 1.0 (purple) for each parameter. Diagrams representing *T. taxirameum* chlorophyll fluorescence are shown. NPQ increases in mosses treated with 10 or 25 μM of metal ions, but decreases at 50 μM, and the color varies largely, therefore not shown in the figure.

Heavy metal majorly affects chloroplast ultrastructure, causing lipid peroxidation in photosynthetic membranes, degrades photosynthetic pigments, inhibits PSII activity and electron transport, decreases carboxylation efficiency of Rubisco and restrains net photosynthetic rate (Mishra and Dubey, 2005). Previous research has suggested that the ratio of Fv/Fm of aquatic moss *Fontinalis antipyretica* could be used as an indicator of heavy metal toxicity (Rau et al., 2007). Other studies have only used chlorophyll fluorescence as one of the test parameters, along with other physiological parameters, to explore the effects of heavy metal stress on moss. Our previous experimental data (Chen et al., 2015a, 2018a) of chlorophyll fluorescence changes in mosses under heavy metal stress are summarized in Figure 1. In our previous report (Chen et al., 2015a), two moss species *Taxiphyllum taxirameum* and *Eurhynchium eustegium* were compared. They showed similar chlorophyll fluorescence images under metal stresses (the same color patterns with slightly different values). However, *T. taxirameum* has larger-area leaves than *E. eustegium*, and therefore is better for observing chlorophyll fluorescence changes. On the other hand, *T. taxirameum* has more branches and forms dense carpet than other hydrophilous moss species, which increases its capacity to concentrate heavy metals from the water. Therefore the heavy metal accumulation ability of *T. taxirameum* was higher than other hydrophilous moss species (Chen et al., 2010, 2015b).

Phenotypes with anthocyanin accumulation pattern and chlorosis pattern and colors and values of chlorophyll fluorescence images of Fv/Fm and Φ_PSII_ could reflect metal species groups and concentrations roughly (Figure 1). Furthermore, the fluorescence color patterns were nearly the same for all concentrations (10, 25, or 50 μM) of the same heavy metal treatments, although the high concentration (50 μM) led to larger declines in Fv/Fm and Φ_PSII_ values (Chen et al., 2015a). The phenotype of non-stressed mosses was bright green. Copper and Zinc (≤50 μM) did not cause apparent chlorosis but caused noticeable anthocyanin accumulation in leaves. Pb and Cr led partial chlorosis and slightly less anthocyanin accumulation. Cd and Hg did not induce observable anthocyanin accumulation, but instead induced partial or complete chlorosis, respectively, in the entire moss, perhaps due to a large amount of damage to moss cells. The color of Fv/Fm and Φ_PSII_ image is uniform blue and green in a control moss, respectively. Then, the color of Fv/Fm and Φ_PSII_ image is blue with sporadic green and green with sporadic yellow for Cu and Zn-treated mosses, respectively. Lead and Chromium treatment forms Fv/Fm image green with sporadic blue and Φ_PSII_ image turns green with sporadic red. The color of Fv/Fm image is green with sporadic red for Cadmium-treated mosses and a large dark area for Mercury-treated mosses as well as Φ_PSII_ image is a large dark area on Cadmium and Mercury treatment. It is interesting to note that the basal tissues show higher Fv/Fm and Φ_PSII_ fluorescence (blue to green colors) than the apical tissues (green to yellow colors); contrastingly, the basal tissues present much lower NPQ fluorescence (red to black colors) than the apical tissues (green to yellow colors; Chen et al., 2015b, 2018a). These differences may suggest that the apical tissues are subjected to more severe damages than the basal tissues during the metal stress. Correspondingly, more superoxide accumulation and more cell death were observed for the apical tissues than the basal tissues (Chen et al., 2015b, 2018a).

The colors changes indicate great reductions in these parameters. For example, 5–20% declines in Fv/Fm and Φ_PSII_ values indicate approximate 10 μM metal ions; 20–40% declines in these two parameters indicate 25 μM metal ions; 40–75% declines indicate 50 μM metal ions. Detailed value changes have been shown in the report by Chen et al. (2015a). Despite the fact that some moss species (like *Physcomitrella*) has a much stronger NPQ than higher plants (Alboresi et al., 2010; Chen et al., 2018b), the NPQ value increases in mosses treated with 10 or 25 μM of metal ions, but decreases at 50 μM, and the color varies largely (therefore not shown in Figure 1). The detection limits are in great variation among different metal species groups. 50 μM Cu, Zn, Pb, or Cr (VI) treatments would result in about half declines in Fv/Fm and Φ_PSII_ values. However, over 70% declines could be observed for 50 μM Cd or Hg ions (a large dark area on the chlorophyll fluorescence image). Therefore, 50 μM is the detection limit for Cd or Hg contaminants (Chen et al., 2015a). While for less toxic metal ions, the detection limits may be up to 500 μM (Chen et al., 2015b, 2018a).

Moreover, we reported the difference of toxicity between trivalent chromium and hexavalent chromium using chlorophyll fluorescence (Chen et al., 2018a), and found that Cr (III) and Cr (VI) could be monitored distinguishably according to the NPQ of sporadic purple and sporadic lavender images, respectively (Figure 1). White-purple spots appear on the edges of the leaves at the apical tissues in the NPQ image for Cr (VI)-treated mosses. As the Cr (VI) treatment concentration increases, white-purple points increasingly begin to appear. However, regardless of the concentration of Cr (III), the NPQ image of Cr (III)-treated mosses is mostly blue-purple patches with no white-purple spots (Chen et al., 2018a). White-purple spots present extremely high NPQ values on these points, also indicating that apical tissues have higher NPQ fluorescence than the basal tissues. Detailed value changes have been shown in the report by Chen et al. (2018a). Our previous report indicated that the uptake of Cr (VI) was much easier than Cr (III) in moss plants (almost two times higher). Thus for less mobile Cr (III) ions, the detection limits may be further up to 1000 μM (Chen et al., 2018a).

Chlorophyll fluorescence appeared to be a useful technique to monitor heavy metals in water or on wetland and humid soil surface without destructive measurements. However, two or more metal contaminants may co-exist in a natural environment. For example, it is also difficult to judge the degree of Cr pollution if other heavy metal contaminants are present. Therefore, this method is suitable for detecting single Cr contaminations in an area known to be polluted by Cr (Chen et al., 2018a).

## Other Abiotic Stresses May Not Affect Heavy Metal Biomonitoring by Moss Chlorophyll Fluorescence

**FIGURE 2:**
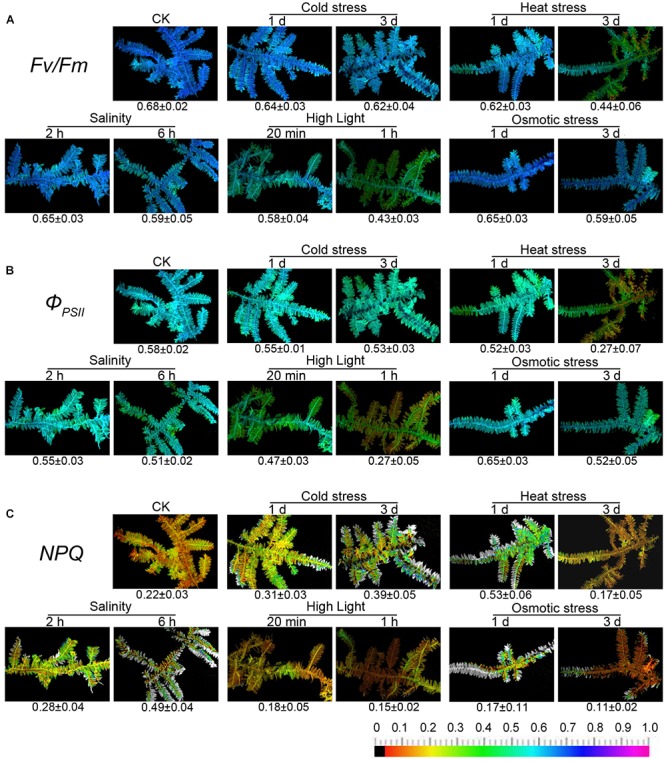
Chlorophyll fluorescence imaging of *T. taxirameum* under cold stress, heat stress, salt stress, high light, and osmotic stress. Experimental materials were *T. taxirameum* (Mitt.) Fleisch, which were collected in Sichuan Agricultural University (Ya’an, China) and brought back to laboratory to be thoroughly washed. *T. taxirameum* were further acclimatized in distilled sterile modified Mohr medium for 3 days under the controlled conditions (16/8 h light/dark cycles at 100 μmol of photons m^-2^ s^-1^, 25 ± 1°C) before stress treatment. Adapted mosses were transferred to 10°C or 45°C 1 day/3 days for cold or heat stress. For salt stress, adapted mosses were placed in 300 mM NaCl solution for 2 or 6 h. For high-light stress, adapted mosses were placed at 1000 μmol of light photons m^-2^ s^-1^, 25 ± 1°C for 20 min or 1 h. Adapted mosses were placed in 20% PEG solution for 1 or 3 days for osmotic stress. Chlorophyll fluorescence images were obtained at room temperature using a modulated imaging fluorometer (the Imaging PAM *M*-Series Chlorophyll Fluorescence System, Heinz-Walz Instruments, Effeltrich, Germany) according to the instructions provided by the manufacturer. Images of Fv/Fm after dark adaptation and of Φ_PSII_ and NPQ at the steady-state of the induction curve with actinic illumination of 100 μmol photons m^-2^ s^-1^. The color scale shows at the bottom of the figure represents the range from 0 (black) to 1.0 (purple) for each parameter. **(A–C)** Means the three chlorophyll fluorescence parameters Fv/Fm, Φ_PSII_, and NPQ respectively.

Despite the well-documented influence of abiotic stress on photosynthetic processes and chlorophyll fluorescence being explored in many higher plants (Kalaji et al., 2016), the relationship between abiotic stress and chlorophyll fluorescence of mosses is weakly summarized in the literature. An experiment was carried out to observe how the chlorophyll fluorescence of moss changed under several common stresses, including cold, heat, salinity, high light, and osmotic stress. The experimental results have been shown in Figure 2.

Only heat stress and high light have a significant effect on the fluorescence parameters of *T. taxirameum* among five stresses. In contrast, *T. taxirameum* is less sensitive to short-term cold, salinity, and osmotic stress as the fluorescence parameters do not change much with the timed exposure. When chlorophyll fluorescence of moss is used to biomonitor heavy metal contamination in aquatic environment, where both heat stress and high light are present at the same time, the fluorescence parameters are seriously reduced, resulting in an inaccurate observation. However, the effects of cold, salinity, and osmotic stress are minimal. It is important to note that the value of NPQ decreases rapidly under osmotic stress, so it should not be possible to distinguish Cr (VI) from Cr (III) by a NPQ image when there is osmotic stress. The fluorescence parameters of mosses are diverse under five stresses, so the detailed mechanism remains to be further studied.

Mosses are usually grown in the shaded (cool) and humid (water) environments, such as the shallow water or the soil at the water’s edge, where heat stress, high light or osmotic stress does not usually co-occur. Alternatively, we should not select mosses grown under high-light conditions when acquiring chlorophyll fluorescence images. Although mosses may encounter low temperature or salinity, these two environmental stresses do not affect the chlorophyll fluorescence significantly. Thus, heat stress, high light, or osmotic stress may not compromise the accuracy of heavy metal biomonitoring through the moss chlorophyll fluorescence.

## Conclusion and Perspectives

Chlorophyll fluorescence tool provides useful information about plant photosynthetic performance and the extent to which this performance is limited by photochemical and non-photochemical processes. As reported in this article, this technique has been conveniently used in stress researches where it provides the possibility to detect the responses of mosses to abiotic stresses. Previous studies provide new ideas to monitor water heavy metal rapidly and non-invasively in a large-scale using moss chlorophyll fluorescence parameters with imaging. However, it is difficult to identify specific stresses in any plant including moss directly through chlorophyll fluorescence tool for the time being. In the laboratory, it is easy to create a single controllable stress condition to induce clear symptoms, whereas in the field, plant is often exposed to several joint stresses at the same time. Nevertheless, mosses are usually grown in the shaded and humid environments without heat stress, high light or osmotic stress (or these environmental stresses can be avoided). And they are insensitive to low temperature or salinity. Thus, other environmental factors may not affect heavy metal biomonitoring by the moss-chlorophyll-fluorescence method.

For highly toxic metal ions (such as Cd and Hg), the detection limit is about 50 μM, contrasting to 500 μM for low toxic metal ions. However, Cd or Hg ion concentrations may exceed 50 μM in some heavily polluted water. How to monitor these metal ions at extremely high levels needs further investigation. Our previous work only considered the case of a certain heavy metal stress, but mosses often suffer many heavy metals at the same time in the real natural environment. Studying the chlorophyll fluorescence of the moss after multiple heavy metals treatments will be the future research direction. In addition, the effect of water organic matter pollution on the chlorophyll fluorescence of moss has not been studied, and it is therefore another research direction for the future. Nevertheless, a single mine water pollution usually contains only one major metal iron. For example, lead mine often contaminates nearby surface water by letting out Pb irons. While chromium occurs naturally as both a chromite (FeCr_2_O_4_) in serpentine and ultramafic rocks and mostly exists in trivalent Cr (Becquer et al., 2003). Our method with the moss chlorophyll fluorescence may be especially useful to monitor heavy metal in the surface water of mining areas. However, for monitoring industrial contaminants (mixtures of toxic organic pollutants and inorganic pollutants including heavy metals), more feasible methods still need to be developed.

Most previous studies of moss chlorophyll fluorescence (Rau et al., 2007; Proctor and Smirnoff, 2011; Liepiņa and Ievinsh, 2013; Kangas et al., 2014; Jägerbrand and Kudo, 2016) focused on the fluorescence value changes. Our former studies (Chen et al., 2015a, 2018a) and the data proposed in this perspective suggest that color patterns of the fluorescence images could reflect metal species groups and concentrations. And the color pattern remains stable as the metal concentration increases. For Fv/Fm and Φ_PSII_, a higher treatment concentration would result in a larger area of colors representing low fluorescence values. For example of Cd treatments, 50 μM led to a larger area of red on Fv/Fm image, than that of 10 μM treatment. However, the color pattern always is “Green with sporadic red,” regardless of the concentration of Cd (Chen et al., 2015a). Metal-iron-specific fluorescence color pattern can only be observed in moss plants, but not in higher plants (data not shown). Different colors on the same moss thallus may indicate different accumulations of metal irons at different parts of the thallus, or possible translocation of metal irons within a thallus, or some particularity of moss chlorophyll fluorescence, which requires further explorations.

## Author Contributions

Y-EC and SY conceived the perspective and wrote the manuscript. NW collected the data. Z-WZ and MY helped to writing the manuscript.

## Conflict of Interest Statement

The authors declare that the research was conducted in the absence of any commercial or financial relationships that could be construed as a potential conflict of interest.
